# Thrombospondin-1 Signaling Through the Calreticulin/LDL Receptor Related Protein 1 Axis: Functions and Possible Roles in Glaucoma

**DOI:** 10.3389/fcell.2022.898772

**Published:** 2022-05-27

**Authors:** Joanne E. Murphy-Ullrich

**Affiliations:** ^1^ Departments of Pathology, University of Alabama at Birmingham, Birmingham, AL, United States; ^2^ Department of Cell Developmental and Integrative Biology, University of Alabama at Birmingham, Birmingham, AL, United States; ^3^ Department of Ophthalmology and Visual Sciences, University of Alabama at Birmingham, Birmingham, AL, United States

**Keywords:** thrombospondin-1, calreticulin, LRP1, glaucoma, focal adhesions, anoikis, matricellular, cell migration

## Abstract

Thrombospondin-1 (TSP-1) is a matricellular extracellular matrix protein. Matricellular proteins are components of the extracellular matrix (ECM) that regulate key cellular functions and impact ECM organization, but which lack direct primary structural roles in the ECM. TSP-1 expression is upregulated in response to injury, hypoxia, growth factor stimulation, inflammation, glucose, and by reactive oxygen species. Relevant to glaucoma, TSP-1 is also a mechanosensitive molecule upregulated by mechanical stretch. TSP-1 expression is increased in ocular remodeling in glaucoma in both the trabecular meshwork and in the optic nerve head. The exact roles of TSP-1 in glaucoma remain to be defined, however. It plays important roles in cell behavior and in ECM remodeling during wound healing, fibrosis, angiogenesis, and in tumorigenesis and metastasis. At the cellular level, TSP-1 can modulate cell adhesion and migration, protease activity, growth factor activity, anoikis resistance, apoptosis, and collagen secretion and matrix assembly and cross-linking. These multiple functions and macromolecular and receptor interactions have been ascribed to specific domains of the TSP-1 molecule. In this review, we will focus on the cell regulatory activities of the TSP-1 N-terminal domain (NTD) sequence that binds to cell surface calreticulin (Calr) and which regulates cell functions via signaling through Calr complexed with LDL receptor related protein 1 (LRP1). We will describe TSP-1 actions mediated through the Calr/LRP1 complex in regulating focal adhesion disassembly and cytoskeletal reorganization, cell motility, anoikis resistance, and induction of collagen secretion and matrix deposition. Finally, we will consider the relevance of these TSP-1 functions to the pathologic remodeling of the ECM in glaucoma.

## Introduction

The extracellular matrix (ECM) is a meshwork of proteins and carbohydrate-rich molecules secreted by cells and organized into distinct cell and context-specific structures in the extracellular space. Cellular interactions with ECM regulate and direct key cellular functions, such as differentiation, survival, proliferation, adhesion, and migration/invasion. In turn, cells both determine the expression and secretion of specific ECM components as well as the organization of the ECM network, a concept termed “dynamic reciprocity” (Bornstein and Ash, 1977; Bissell et al., 1982; Bissell and Aggeler, 1987; Bissell and Barcellos-Hoff, 1987). Multi-cellular life depends on ECM. New approaches that enrich ECM components in samples for mass spectrometry/proteomic analyses have increased our understanding of the breadth of molecules contained in the “matrisome” and importantly, enabled comparative studies to assess how the composition of the ECM differs in health and disease (Naba et al., 2012; Naba et al., 2016). These studies have identified over 278 core matrisome components consisting of various collagens, proteoglycans, and modular glycoproteins in addition to matrisome-associated components consisting of proteases, cross-linking enzymes, growth factors, and glycan structures (Naba et al., 2016).

The eye is an ECM-rich organ. For example, the vitreous, cornea, sclera, and lamina cribrosa are collagen rich structures with distinct tissue-specific collagen type expression and organization. Type IV collagen rich basement membranes are key to integrity of the retinal vasculature and the trabecular meshwork. Thus, it is not surprising that aberrant remodeling of the ECM is critical to the pathogenesis of multiple ocular diseases, including myopia, cataract formation and fibrous encapsulation, age-related macular degeneration, and importantly, glaucoma (Hernandez, 1993; Yang et al., 1993; Kantorow et al., 2000; Norose et al., 2000; Sawhney, 2002; Yan et al., 2003; Mansergh et al., 2004; Rada et al., 2006; He et al., 2014; Kuchtey and Kuchtey, 2014; Biasella et al., 2020; Wu et al., 2020; Grillo et al., 2021; Reinhard et al., 2021; Keller and Peters, 2022; Powell et al., 2022).

## Extracellular Matrix and Glaucoma

Glaucoma is the second leading cause of irreversible blindness as a result of damage to the optic nerve (Quigley, 2011; Tham et al., 2014). Elevated intraocular pressure (IOP) is the most predominant risk factor for glaucoma, although there are other genetic factors that can cause glaucoma and patients with normal intraocular pressures can also develop glaucoma (Downs et al., 2011; Quigley, 2011). In addition, glucocorticoid treatment can lead to glaucoma (Chan et al., 2019; Roberti et al., 2020). Intraocular pressure is maintained by a balance of aqueous humor production and its outflow primarily through the conventional trabecular meshwork (TM) pathway in the anterior of the eye (Acott et al., 2021). Complex remodeling of diverse ECM components is linked to increased outflow resistance in the TM and elevated IOP (Vranka et al., 2015a; Vranka et al., 2015b; Acott et al., 2021; Keller and Peters, 2022). Chronic IOP can lead to deformation of the collagenous lamina cribrosa (LC) and adjacent sclera at the posterior of the eye: cupping of the LC is associated with axonal damage to the retinal ganglion cells of the optic nerve (Downs et al., 2011). Abnormal elastin fibers, increases in matrix metalloproteinases, and altered collagen organization in the laminar beams and at the posterior laminar boundary are among the ECM changes in the glaucomatous LC (Hernandez et al., 1990; Pena et al., 1998). Remodeling of the ECM affects the structure and function of both the TM and LC and it is accepted that deleterious ECM remodeling is a significant factor in the pathogenesis of glaucoma (Hernandez et al., 1990; Hernandez, 1993; Lutjen-Drecoll, 1999; Tektas and Lutjen-Drecoll, 2009; Vranka et al., 2015b; Wang et al., 2017; Kelly et al., 2021). Matricellular ECM proteins, a distinct type of ECM protein described below, are widely considered to be important in the pathogenesis of glaucoma [reviewed in (Rhee et al., 2009; Chatterjee et al., 2014; Wallace et al., 2014)].

## Matricellular Proteins

Distinct from ECM proteins that comprise the primary structure of the ECM and that support cell adhesion, such as collagens, laminins, and fibronectin, there is another group of ECM proteins, matricellular proteins, that instead modify ECM structure and organization and which are primarily de-adhesive (Sage and Bornstein, 1991; Bornstein, 1995; Murphy-Ullrich and Sage, 2014). Common to both matricellular and structural ECM components, matricellular proteins bind to diverse cellular receptors to regulate cellular functions. In addition, they also bind to multiple other ECM components and modulate growth factor and protease activity. In the mid-1990’s when the first knockout mice of matricellular proteins, such as tenascin-C, SPARC, and TSP-1, were generated, there was surprise in the field that these genetic knockouts did not result in embryonic lethality. However, distinct phenotypes became apparent when animals were subjected to stresses, aging, or injury, suggesting important roles for matricellular proteins in adaptive and pathologic responses to injury and stress (Erickson, 1997; Settles et al., 1997; Lawler et al., 1998; Bassuk et al., 1999; Brekken and Sage, 2000; Bradshaw et al., 2002; Bradshaw et al., 2003a; Bradshaw et al., 2003b; Haddadin et al., 2009). These roles of matricellular proteins in adaptive cellular responses have been borne out by subsequent studies from multiple labs and are consistent with the highly regulated patterns of matricellular protein expression during development, following injury, and under conditions of cellular and metabolic stress (Chen et al., 2000; Adams and Lawler, 2011; Stenina-Adognravi, 2014; Giblin and Midwood, 2015; Midwood et al., 2016; Zuliani-Alvarez et al., 2017). The number of ECM proteins that are now considered to be “matricellular” has also expanded since the first insights with TSP-1, tenascin-C, and SPARC (Murphy-Ullrich and Sage, 2014). Myocilin, a protein expressed in glaucoma, has long been considered a matricellular protein because of its de-adhesive effects on TM cells and interactions with multiple ECM proteins (Tamm, 2002; Wentz-Hunter et al., 2004; Shen et al., 2008; Chatterjee et al., 2014).

## Thrombospondin-1

**FIGURE 1:**
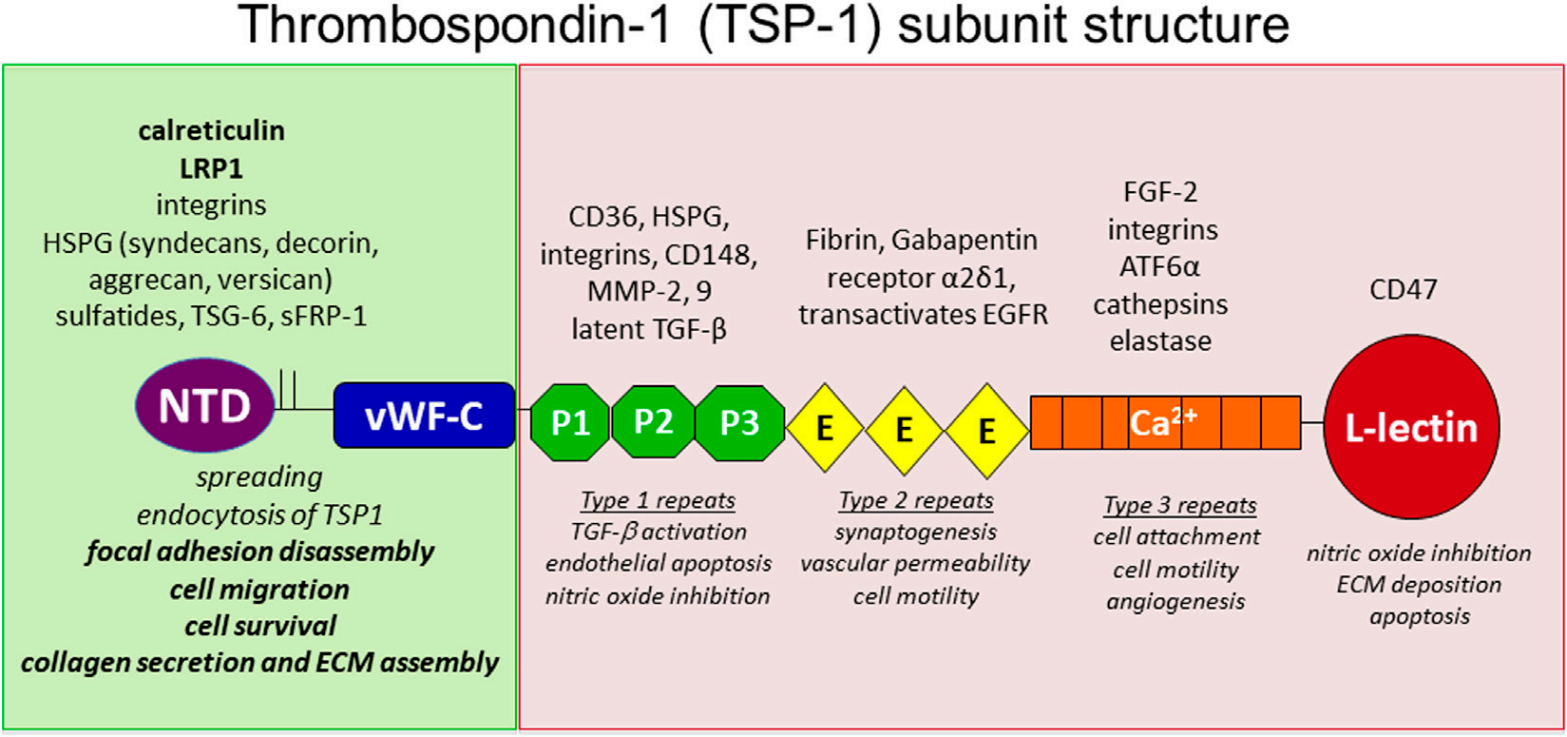
Model of Thrombospondin-1. TSP-1 is a homotrimer linked by interchain disulfide bonds in the oligomerization domain between the laminin G like N-terminal domain (NTD) and the vWF-C like domain. It is comprised of 3 properdin-like repeats (also called TSR or Type 1 repeats), followed by 3 EGF-like repeats (Type 2 repeats). This is followed by 13 calcium-binding Type 3 repeats that interact with the C-terminal L-lectin like domain. Various receptors and interacting molecules for each domain are indicated above the structure and functions ascribed to each domain are indicated below the structure.

Here, we will highlight the matricellular ECM glycoprotein, thrombospondin-1 (TSP-1), encoded by *THBS1,* and discuss possible roles for TSP-1 signaling of intermediate adhesion and its associated functions in glaucoma. TSP-1 is a homotrimeric, modular ECM glycoprotein that is classified as a matricellular ECM protein. TSP-1 was first identified as a protein released from the α-granules of thrombin-stimulated platelets and subsequently found to be widely expressed by cells in culture. TSP-1 is part of a family of five related proteins, with trimeric TSP-1 and TSP-2, members of the Group A TSP family, being the most similar [reviewed in (Adams and Lawler, 2011)]. Typical of ECM proteins, TSP-1 is a modular glycoprotein composed of multiple repeats of distinct structural domains. TSP-1 interacts with diverse molecules, including other ECM proteins, proteases, and growth factors, and it has multiple receptors, imparting an array of functionalities in different contexts (Adams and Lawler, 2011; Murphy-Ullrich and Sage, 2014; Resovi et al., 2014) (Figure 1). TSP-1 has many biologic functions, including regulation of cell adhesion and motility, platelet aggregation, cell death, protease activity, ECM organization, and growth factor regulation. Given this multiplicity of functions, TSP-1 has diverse roles in regulation of angiogenesis, inhibition of nitric oxide signaling, endothelial cell barrier function, inflammation/immunity, endoplasmic reticulum stress, fibrosis, and latent TGF-β activation (Adams and Lawler, 2004; Sweetwyne and Murphy-Ullrich, 2012; Murphy-Ullrich and Suto, 2018; Murphy-Ullrich, 2019; Forbes et al., 2021; Kale et al., 2021; Kaur et al., 2021; Morandi et al., 2021). The reader is referred to several excellent reviews covering broader roles of TSP-1 and other matricellular proteins in glaucoma and in fibrotic ECM remodeling for a more extensive discussion (Rhee et al., 2009; Chatterjee et al., 2014; Wallace et al., 2014; Murphy-Ullrich and Downs, 2015; Murphy-Ullrich, 2019; Keller and Peters, 2022). In this review, we will focus specifically on the N-terminal domain (NTD) calreticulin (Calr) binding sequence of TSP-1. This sequence, mimicked by the “hep I” peptide, signals induction of the intermediate cell adhesive state, first associated with the de-adhesive activity of TSP-1 (Murphy-Ullrich et al., 1993).

## Thrombospondin-1 Regulation of Intermediate Cell Adhesion

### Characterization of the Intermediate Adhesive State: Altered Focal Adhesion and Cytoskeletal Organization

Matricellular proteins are distinct from adhesion supporting ECM proteins such as fibronectin, in that although cells might attach and partially spread on substrates of matricellular proteins, cells do not undergo complete cytoskeletal organization indicative of a fully adhesive cell (Lahav et al., 1987; Tuszynski et al., 1987; Murphy-Ullrich and Hook, 1989; Chiquet-Ehrismann, 1991; Adams and Lawler, 1993). It was originally noted that cells attached to substrates of TSP-1 had limited spreading with the formation of fascin-containing microspikes and also the absence of central focal adhesions and actin stress fiber organization (Murphy-Ullrich and Hook, 1989; Adams, 1995; Adams et al., 2001). In addition, soluble TSP-1 can prevent bovine aortic endothelial cells from forming focal adhesions on fibronectin substrates (Murphy-Ullrich and Hook, 1989). Similarly, tenascin-C can prevent cell adhesion to fibronectin substrates by blocking fibronectin-syndecan-4 binding (Huang et al., 2001; Midwood et al., 2004; Midwood et al., 2006). Moreover, TSP-1 and other matricellular proteins can induce alterations in fully adherent cells containing organized F-actin containing stress fibers and focal adhesions, characterized by disassembly of existing focal adhesions, but not cell detachment, a state called “intermediate cell adhesion” (Greenwood and Murphy-Ullrich, 1998; Murphy-Ullrich, 2001). Intermediate adhesion refers to this altered adhesive state in which cells are poised to respond to stress through altering their cytoskeletal organization and activation of signaling pathways that stimulate cell motility, survival, and ECM organization. These observations were first made in studies with SPARC, tenascin-C, and TSP-1 (Murphy-Ullrich and Hook, 1989; Murphy-Ullrich et al., 1991; Sage and Bornstein, 1991; Murphy-Ullrich et al., 1995). Mesenchymal cells (endothelial cells, fibroblasts) adherent to substrates such as fibronectin or vitronectin when treated with soluble forms of these matricellular proteins undergo cytoskeletal reorganization with loss of vinculin and α-actinin from focal adhesions at the termini of actin stress fibers. Interestingly, cells remain attached and partially spread. Furthermore, integrin remains clustered at these regions, suggesting that matricellular proteins are triggering intracellular signaling events that cause cytoskeletal reorganization, rather than direct physical disruption of integrin-ECM interactions (Murphy-Ullrich, 2001). The upregulation of TSP-1 expression in cell injury and in response to multiple forms of cellular stress, including hypoxia, mechanical stimulation, and high glucose, among others, supports the idea that TSP-1 signaling of intermediate adhesion might be a cell adaptive response (Stenina-Adognravi, 2014).

### The Calreticulin/LRP1 Receptor Co-Complex Mediates Thrombospondin-1 Induced Intermediate Adhesion

The N-terminal heparin binding domain of TSP-1, specifically amino acids 17–35, binds to a calcium binding protein, calreticulin (Calr), to mediate focal adhesion disassembly and this function of TSP-1 can be mimicked by a peptide comprising this sequence, hep I (Murphy-Ullrich et al., 1993; Goicoechea et al., 2000). Notably, this sequence and function are conserved in TSP-2, although direct binding of TSP-2 to Calr has not been experimentally confirmed. The TSP binding site in Calr has been mapped to a sequence in the Calr N-domain (aa 19–36) on the face opposite Calr’s lectin binding site (Goicoechea et al., 2002). Calr is a chaperone involved in mediating glycoprotein folding in the endoplasmic reticulum (ER) (Michalak et al., 2009). It also is a calcium binding protein that mediates calcium release from the ER leading to downstream NFAT activation (Michalak et al., 1999; Groenendyk et al., 2004). However, Calr is found in multiple different cellular compartments, including the juxtamembrane cytosol where it interacts with integrin alpha subunits and also at the cell membrane on the cell surface (Dedhar, 1994; Coppolino and Dedhar, 1999; Gold et al., 2010; Liu et al., 2016). It is not clear how Calr is transported and localized to the cell surface, although conditions of cellular stress increase cell surface expression of Calr and there is evidence to suggest that Calr is transported with phosphatidyl serine to the surface of apoptotic cells (Wiersma et al., 2015). Integrin expression also regulates transit of Calr to the cell surface (Liu et al., 2016).

In addition to mediating TSP-1 induced focal adhesion disassembly, migration, anoikis, and collagen matrix assembly (see below), cell surface Calr, together with LDL Receptor Related Protein 1, (LRP1), is an important component of immunogenic cell death, serving as a cell surface signal for phagocytosis, independent of ligation by TSP-1 (Ogden et al., 2001; Gardai et al., 2005; Obeid et al., 2007). Secreted, extracellular Calr (eCRT) also facilitates scarless wound healing primarily via binding the scavenger and signaling receptor, LRP1 (discussed below), which leads to release of TGF-β3 and autocrine signaling to induce expression of ECM proteins (Nanney et al., 2008; Greives et al., 2012; Pandya et al., 2020). Interestingly, the ability of eCRT to induce ECM is dependent on intracellular Calr, consistent with previous observations showing the importance of endoplasmic Calr mediated calcium release and NFAT activation in mediating TGF-β stimulation of ECM (Van Duyn Graham et al., 2010; Zimmerman et al., 2013; Zimmerman et al., 2015; Owusu et al., 2018; Lu et al., 2020). Whether TSP-1 binding to cell surface Calr and complex formation with LRP1 has a role in this process remains unknown. In addition, Calr on the surface of the parasite *Trypanisoma cruzi* (TcCRT) binds TSP-1 and recombinant NTD, and localizes TSP-1 to the surface of the parasite, which increases parasite infectivity of mammalian cells (Johnson et al., 2012; Nde et al., 2012).

Since Calr is a peripheral membrane protein that lacks a transmembrane domain, Calr must associate with a transmembrane molecule to transmit intracellular signaling resulting from TSP-1 ligation of cell surface Calr. Basu and Srivastava previously showed an association between cell surface Calr and CD91, otherwise known as LRP1 or the α_2_-macroglobulin receptor (Strickland et al., 1995; Basu and Srivastava, 1999; Basu et al., 2001; Lillis et al., 2008). LRP1 is a large, multi-domain protein that binds an array of ligands. It is comprised of two disulfide-linked proteins, the smaller of which traverses the cell membrane (Strickland et al., 1995). The cytoplasmic domain of LRP-1 contains NPXY-1 sites that are involved in mediating intracellular signal transduction from bound ligand (Lillis et al., 2008). LRP1 mediates TSP-1 stimulated focal adhesion disassembly, cell migration, and anoikis resistance (Orr et al, 2003a; Orr et al, 2003b; Pallero et al., 2008). It was previously shown that TSP-1 binds to LRP1 and that LRP1 is a major mediator of endocytosis of TSP-1, a process enhanced by heparan sulfate proteoglycans (Godyna et al., 1995; Mikhailenko et al., 1995; Mikhailenko et al., 1997; Wang et al., 2004). This LRP1 binding site is localized to the N-terminal domain of TSP-1 (aa 1–90), although this site is distinct from the hep I site (aa 17–35) that binds Calr and mediates focal adhesion disassembly (Goicoechea et al., 2000; Wang et al., 2004). Furthermore, there is no evidence that the Calr-binding site of TSP-1 binds LRP1 (Orr et al, 2003b). LRP1 mediated endocytosis of TSP-1 could be an important factor in limiting TSP-1 action in a localized cellular milieu and there is also evidence suggesting that this endocytic role of LRP1 is involved in clearance of TSP-1/TSP-2 ligands such as MMPs and VEGF [discussed in (Lillis et al., 2008)].

It should be noted that different matricellular proteins signal intermediate adhesion *via* distinct receptors. For example, the large form of tenascin-C containing the alternatively spliced TNfnA-D domain binds to annexin II to trigger focal adhesion disassembly (Chung et al., 1996), whereas, the active receptor for SPARC-mediated focal adhesion disassembly has not been identified.

### Signal Transduction Pathways in Intermediate Adhesion

**FIGURE 2:**
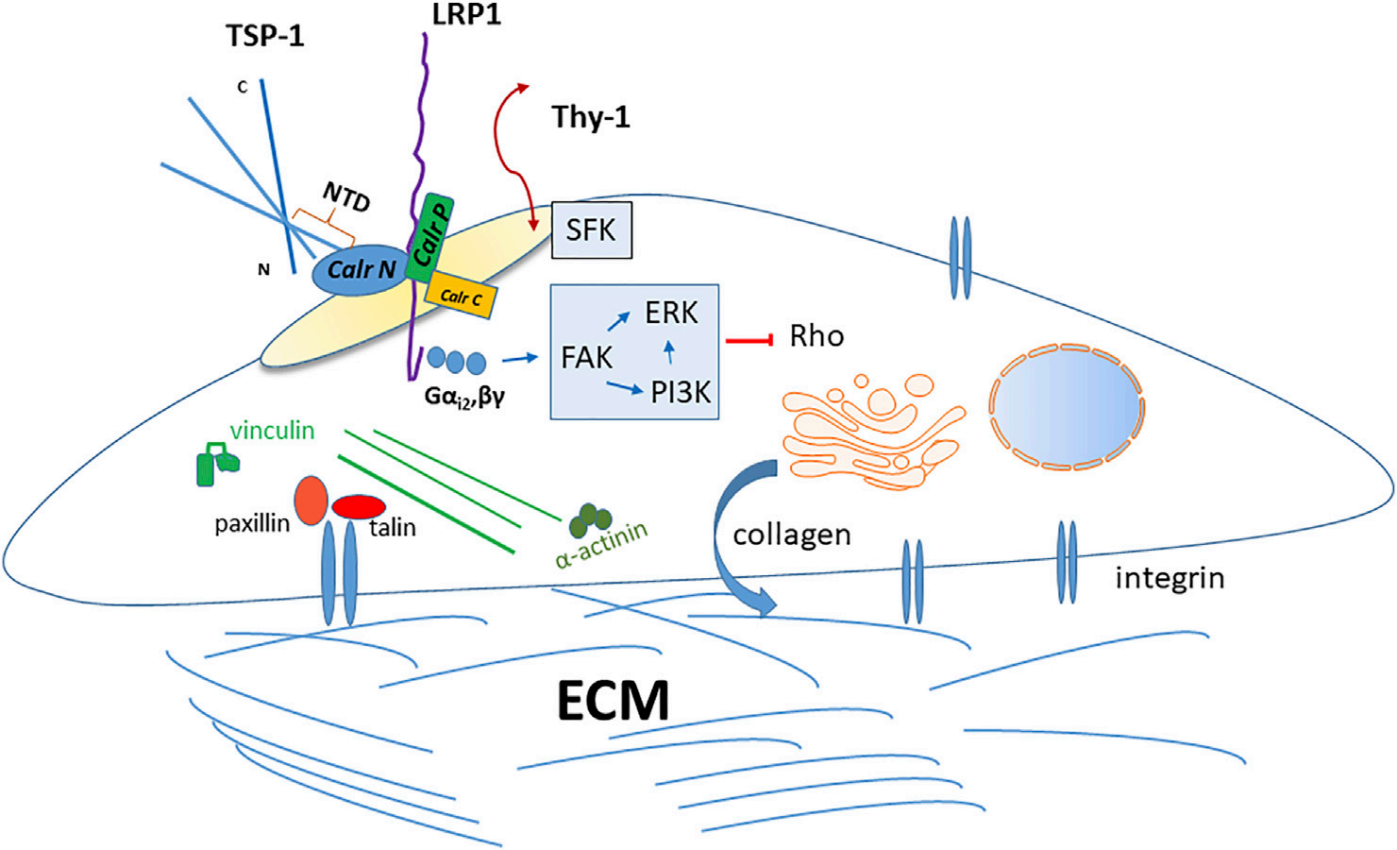
Thrombospondin-1 signaling through the caleticulin-LRP1 complex. The sequence represented by the hep I peptide (amino acids 17–35) in the NTD of TSP-1 bind to the N-terminal domain of calreticulin at the cell surface. The TSP-1 binding site has been localized to amino acids 19–26 in calreticulin. Binding of TSP-1 to calreticulin induces altered interactions between the calreticulin N and P domains to expose the putative LRP1 binding site and increase association of calreticulin with LRP1 and activation of downstream signaling. This occurs in lipids rafts. The GPI-linked protein, Thy-1, must also be present in lipid rafts and it activates Src family kinases, which are necessary for TSP-1 signaling through calreticulin/LRP-1, although direct interactions of Thy-1 with this complex have not been demonstrated. TSP-1-calreticulin engagement of LRP1 induces association with the Gα_i2_ heterotrimeric G protein subunit. This activates downstream FAK that induces PI3K and ERK signaling, culminating in a transient downregulation of Rho kinase activity. In fibroblasts and endothelial cells, TSP-1 signaling through calreticulin/LRP1 induces focal adhesion disassembly with cytoskeletal reorganization accompanied by loss of vinculin and α-actinin from integrin-talin clusters. This state of intermediate adhesion renders cells more motile and resistant to anoikis in a PI3K-dependent manner. *In vivo* expression of the secreted TSP-1 calreticulin binding sequence in a model of the foreign body response increases pericapsular collagen density and increased collagen matrix assembly *in vitro*, similarly in a PI3K-dependent manner.

Greenwood et al. (1998), Greenwood and Murphy-Ullrich (1998) showed that TSP-1 or the hep I peptide stimulated increased PI3K activity and binding of PIP3 to α-actinin, an actin bundling protein, inducing α-actinin dissociation from cytoplasmic focal adhesion complexes. Separate studies using PDGF showed that PIP3 induces α-actinin dissociation from the cytoplasmic tail of the integrin beta subunit and it is presumed that TSP-1 acts similarly (Greenwood et al., 2000). TSP-1 activates FAK, PI3K, and ERK in a manner involving both Gα_i2_ and Gβγ heterotrimeric G protein subunits (Orr et al., 2002). TSP-1 mediated focal adhesion disassembly requires FAK activation, which induces both PI3K and ERK activity that results in a transient down regulation of Rho activity (Orr et al., 2002; Orr et al., 2004). Furthermore, both TSP-1 and tenascin-C stimulation of focal adhesion disassembly requires Protein Kinase G activity (Murphy-Ullrich et al., 1996). Interestingly, Barker et al. (2004b) showed that the GPI-linked protein, Thy-1 (CD90), present in lipid rafts, is required for src family kinase (SFK) activation and TSP-1/hep I-mediated focal adhesion disassembly: both intact lipid rafts and the GPI-anchor of Thy-1 are required for responsiveness to TSP-1/hep I (Barker et al., 2004a; Barker et al., 2004b; Rege et al., 2006). Further work by Rege et al. (2006) showed that Thy-1 associates with FAK and SFK in lipid rafts following TSP-1/hep I stimulation of fibroblasts to induce cell migration and focal adhesion disassembly. There is no evidence for a direct association between Thy-1 and either LRP1 or Calr (Barker et al., 2004b), although both Calr and LRP1 have been localized to lipid rafts (Boucher et al., 2002; Ghiran et al., 2003). These intriguing results suggest a role for Thy-1 in potentially mediating formation of multi-factor signaling complexes associated with Calr/LRP1 following TSP-1/hep I stimulation (Rege et al., 2006). The signaling pathways activated by TSP-1 binding to the Calr/LRP-1 co-complex are summarized in Figure 2.

### Role of Lipid Rafts in Thrombospondin-1/Calr/LRP1 Signaling

Lipids rafts are important in mediating a number of complex effects of various TSP-1 receptors (Morandi et al., 2021). The importance of lipid rafts in mediating TSP-1 interactions with Calr/LRP1 complexes was shown in a series of elegant studies using molecular dynamics simulation approaches (Wang et al., 2014; Wang et al., 2019; Yan et al., 2010; Yan et al., 2011). In initial studies, Yan et al. (2010) showed that TSP-1 binding to the Calr N-terminal domain amino acids 19–36 induced conformational changes in Calr. TSP-1 binding to Calr induced a more open interaction between the Calr N domain and the P domain loop, potentially exposing the LRP1 binding site in Calr. This is consistent with biochemical observations that treatment of cells with the TSP-1 hep I peptide induced increased co-immunoprecipitation of Calr with LRP1 in endothelial cells (Orr et al, 2003b). Further modeling studies confirmed the importance of lysines 24 and 32 in TSP-1 and amino acid 24–26 and 32–34 in Calr for these interactions, consistent with biochemical observations (Murphy-Ullrich et al., 1993; Goicoechea et al., 2002; Yan et al., 2011). The Song lab then modeled the role of various membrane lipid configurations to explore the role of membrane domains in mediating Calr interactions with the plasma membrane and the effect on TSP-1 binding. A raft-like lipid bilayer (containing cholesterol) stabilized Calr binding to the lipid as compared to a palmitoyl phosphocholine bilayer and increased TSP-1 induced Calr conformational change to a more open configuration (Wang et al., 2014). Sphingomyelin in the cholesterol containing raft enhanced these interactions and TSP-1 binding to Calr further induced redistribution of cholesterol into raft-like aggregates (Wang et al., 2014). Furthermore, modeling of accessible sites in raft-bound opened Calr led to the hypothesis that Calr amino acids 96–150 near the N and P domain junction potentially represent the Calr-LRP1 binding site, although this remains to be confirmed biochemically (Wang et al., 2014). Calr-LRP1 interactions on apoptotic cells trigger phagocytosis (Ogden et al., 2001; Gardai et al., 2005) and play a role in immunogenic cell death (Obeid et al., 2007). This process is independent of TSP-1 or hep I binding to Calr, however (Gardai et al., 2005). Interestingly, modeling of Calr-LRP1 interactions in a phosphatidylserine rich lipid raft, representing apoptotic cells, showed that phosphatidyl serine rafts increased Calr membrane binding and interactions with LRP1 by increasing Calr stability at the apoptotic membrane (Wang et al., 2014; Wang et al., 2019). However, interactions of Calr bound to apoptotic membranes with TSP-1 showed reduced favorability of binding as calculated by free binding energy (Wang et al., 2014; Wang and Song, 2020), perhaps accounting for the TSP-1 independence of Calr-LRP1 mediated clearance of apoptotic cells (Gardai et al., 2005). This finding could also suggest decreased responsiveness to TSP-1 induced cellular migration and anoikis resistance under apoptotic conditions.

## Functional Roles of Thrombospondin-1 Signaling Through Calr/LRP1 Complexes

Initial studies regarding TSP-1 signaling through Calr/LRP1 complexes were focused on focal adhesion disassembly and induction of intermediate cell adhesion, a potentially biologically adaptive state between rounded, weakly adherent cells and stationary, non-migratory cells with fully organized stress fibers and focal adhesion complexes (Greenwood and Murphy-Ullrich, 1998; Murphy-Ullrich, 2001). The characteristics of this adhesion modulation were described above. In addition to the direct effects of the TSP-1/Calr/LRP1 pathway on focal adhesion disassembly and cytoskeletal reorganization, this pathway has been shown to impact several biological functions of cells, including cell migration, resistance to anoikis, and enhancement of type 1 collagen secretion and matrix assembly. These functions might be the direct result of focal adhesion disassembly (i.e., migration) or more complex indirect effects of signaling pathways activated through Calr/LRP1 signaling (i.e., anoikis resistance, collagen secretion, and assembly) in the intermediate adhesive state. The impact of TSP-1 signaling through Calr/LRP1 on each of these cellular activities will be discussed.

### Cell Migration

The ability of individual cells to migrate requires fine coordination between adhesive, protrusive, and contractile forces (Lauffenburger and Horwitz, 1996; Webb et al., 2002). TSP-1 has long been known to support cell migration, from haptotaxis on TSP-1 substrates through its C-terminal domain (Taraboletti et al., 1987) to stimulation of chemotaxis by the heparin-binding domain of soluble TSP-1 (Taraboletti et al., 1987; Mansfield et al., 1990; Taraboletti et al., 1990; Mansfield and Suchard, 1994). Given the ability of the Calr binding sequence of the heparin-binding domain of TSP-1 to stimulate focal adhesion disassembly (Murphy-Ullrich and Hook, 1989; Murphy-Ullrich et al., 1993), we asked whether TSP-1 and a peptide representing the Calr-binding sequence, hep I, stimulated directed and random cell migration (Orr et al, 2003a). In transwell assays, both TSP-1 and the hep I peptide, but not an inactive modified hep I peptide, stimulated directed (chemotactic) and random (cytokinesis) migration of bovine aortic endothelial cells at concentrations similar to those required to induce focal adhesion disassembly (Orr et al, 2003a). We observed increased directed cell migration with TSP-1 or hep I under chemotactic gradients. Under chemokinetic conditions, TSP-1 or hep I increased the proportion of cell migrating and migration speed. This migratory response to TSP-1 or hep I was absent in either Calr or LRP1 knockout fibroblasts or in wildtype fibroblasts treated with the LRP1 antagonist, RAP. Treatment with TSP-1 or hep I increased cell migration to a gradient of acidic FGF, whereas TSP-1/hep I reduced basic FGF directed migration (Orr et al, 2003a). Interestingly, TSP-1 had minimal chemoattractant effects on TM cells in a modified Boyden chamber assay, although it is not clear whether migrated cells were able to attach to the membranes in the presence of TSP-1 (Hogg et al., 1995). In our transwell assays with bovine aortic endothelial cells, it was necessary to coat membranes with a mix of fibronectin and vitronectin to support cell attachment of migrated cells in the presence of TSP-1 in the lower wells (Orr et al, 2003a). The TSP-1 binding sequence of Calr (aa 19–36) can also stimulate T cell motility through a 3D type 1 collagen ECM and endogenous TSP-1/cell surface Calr induced T cell motility *via* CD47, a receptor for the C-terminal domain of TSP-1; interestingly, in these studies, the Calr binding sequence of TSP-1 (hep I peptide) blocked T cell motility, presumably by competing with endogenous TSP-1/Calr (Li et al., 2005). These data suggest that signaling through the hep I sequence of TSP-1 binding to Calr in complex with LRP1 potentially impacts tissue remodeling through regulating cell adhesion and migration.

### Anoikis Resistance

Non-hematologic cells require interactions with ECM molecules to generate pro-survival, anti-apoptotic signals (Meredith et al., 1993; Frisch and Francis, 1994). Signaling through integrins is a primary mediator of pro-survival signaling, although in some cases, integrin signaling is not sufficient (Zahir et al., 2003). PI3K induced Akt activation and FAK signaling are primary survival pathways regulated by cell-ECM adhesion (Xia et al., 2004; Yamaki et al., 2007). When cells are deprived of these cell adhesion survival signals, they undergo a form of apoptosis termed “anoikis,” derived from the Greek for “homeless wanderer” (Frisch and Francis, 1994). Interestingly, Rho-mediated cytoskeletal tension is an important mediator of anoikis (Yamaki et al., 2007). Anoikis is important in tissue remodeling during development and in response to injury (Frisch and Francis, 1994). Failure of anoikis impedes resolution of wound healing and can lead to fibrosis through fibroblast persistence (Horowitz et al., 2007). Tumor cell adhesion-independence, meaning survival in the absence of cell-matrix interactions, is a form of anoikis resistance and facilitates tumor metastasis (Liotta and Kohn, 2004).

TSP-1 signaling through binding of its type 1 repeats to CD36 and VEGF antagonism, binding of the C-terminal domain to CD47, as well as inhibition of basic FGF signaling *via* binding of the TSP-1 type 3 repeats, and the involvement of integrins, have been shown to induce endothelial apoptosis and this plays an important role in the anti-angiogenic function of TSP-1 (Good et al., 1990; Taraboletti et al., 1990; Tolsma et al., 1993; Streit et al., 1999; Cursiefen et al., 2004; Isenberg et al., 2009; Sun et al., 2009; Lawler and Lawler, 2012; Rusnati et al., 2019; Morandi et al., 2021). However, the NTD of TSP-1 is known to function differently from its C-terminal regions (Elzie and Murphy-Ullrich, 2004). The NTD heparin binding domain supports endothelial cell proliferation and angiogenesis through both syndecan-4, a transmembrane heparan sulfate proteoglycan, and integrin signaling (Tolsma et al., 1993; Ferrari do Outeiro-Bernstein et al., 2002; Calzada et al., 2003; Nunes et al., 2008). Given that the hep I Calr binding sequence in the TSP-1 NTD activates two pro-survival pathways, PI3K and FAK signaling, and transiently down regulates Rho activity that is necessary for anoikis, we asked whether TSP-1 binding to the Calr-LRP1 co-complex might support cell survival under adhesion-deprived conditions that trigger anoikis (Greenwood et al., 1998; Orr et al., 2004; Pallero et al., 2008). Fibroblasts plated on poly-HEMA coated coverslips, which only weakly supports attachment, undergo apoptosis. Treatment with TSP-1, hep I peptide, or a recombinant form of the NTD trimer (NoC1) increased cell viability and prevented apoptosis with decreased Caspase 3 activity and cleaved PARP1. TSP-1/hep I/NoC1 mediated cell survival is dependent on PI3K activity and Akt activity as the survival effects are blocked by wortmannin. Furthermore, TSP-1 mediated anoikis resistance occurs through Calr/LRP1 signaling as a peptide that blocks TSP-1 binding to Calr (CRT19.36), expression of Calr mutated in the TSP-1 binding site, and knockout of LRP1 expression all prevent TSP-1’s pro-survival signaling during anoikis (Pallero et al., 2008). These findings suggest a possible role for TSP-1/Calr/LRP1 signaling in wound repair with early anoikis resistance potentially supporting initial cell survival during migration into wounds, but possibly detrimental effects during later wound resolution due to fibroblast persistence, potentially contributing to fibrosis.

### Collagen Matrix Assembly and the Foreign Body Response

To begin to assess possible roles of TSP-1 signaling through the Calr-LRP1 complex *in vivo*, we used a model of the foreign body response in which surgical sponges loaded with type 1 collagen and plasmid to express a secreted, EGFP-tagged version of the TSP-1 Calr binding sequence are implanted subcutaneously (Sweetwyne et al., 2010). In this model, cells invade the sponge and ingest the collagen and plasmid, becoming locally transfected to express the secreted TSP-1 Calr binding sequence. Given the known roles of this TSP-1 sequence in supporting cell migration and survival and the observations of delayed granulation tissue in *Thbs1* null excisional wounds, we hypothesized that local expression of the TSP-1 Calr binding sequence would augment cell migration and wound healing (DiPietro et al., 1996; Agah et al., 2002; Orr et al, 2003a; Pallero et al., 2008). Surprisingly sponges with local TSP-1 Calr expression, but not control sponges expressing TSP-1 mutated in Calr binding sequences, developed a robust collagenous capsule surrounding the foreign body. This could not be explained by increased cellular or myofibroblast infiltration. Further *in vitro* studies established that the TSP-1 Calr sequence directly increased intracellular collagen expression, soluble collagen secretion, and collagen deposition into a deoxycholate insoluble matrix (Sweetwyne et al., 2010). The effects of the hep I sequence on fibronectin matrix assembly are not known. This increase in collagen matrix is dependent of Akt signaling, but independent of TGF-β signaling. Although the specific mechanism of TSP-1/Calr signaling increased collagen matrix assembly is not clear yet, it does not involve alterations in type 1 collagen transcript or processing of its N and C-terminal propeptides. We do not think that TSP-1/Calr/LRP-1 signaling affects MMP-mediated tissue remodeling by preventing or inhibiting LRP1-mediated MMP clearance as we failed to observe any differences in MMP activity in the presence of hep I peptide (Bing Su, Mariya Sweetwyne, Joanne Murphy-Ullrich, unpublished data). Although the specific roles of TSP-1 signaling through the Calr-LRP1 axis in normal and pathologic wound repair and tissue remodeling remain to be determined, these data provide support for TSP-1/Calr/LRP1 mediated signaling in regulating tissue repair responses (Sweetwyne and Murphy-Ullrich, 2012). Intriguing studies showed that TSP-1, *via* an unknown binding domain, can bind to both the KGHR sequence in the mature triple helical domain of collagen and to the C-propeptide domain of intracellular collagen and directly affect collagen fibril formation by preventing BMP-1 activation of prolysyl oxidase (Rosini et al., 2018). Peptides that block the TSP-1/KGHR interaction increase myofibroblasts through TGF-β signaling, suggesting a complex role for TSP-1 in regulating collagen processing and secretion and ECM assembly through multiple mechanisms.

## Relevance to Glaucoma

So what role might these functions of the NTD of TSP-1 play in the pathologic changes to ocular tissue structure in glaucoma? Multiple studies suggest that TSP-1 expression is variably increased in the glaucomatous TM and in the (LC of the optic nerve head and sclera at the posterior of the eye (Tripathi et al., 1991; Hiscott et al., 1996; Flugel-Koch et al., 2004; Rhee et al., 2009; Chatterjee et al., 2014; Semba et al., 2021). Furthermore, knockout of the *Thbs1* gene in mice reduces intra-ocular pressure (Haddadin et al., 2012). Recently a new gene variant of *THBS1* was identified in a family with primary open angle glaucoma with elevated intraocular pressure that encodes the known N700S missense mutation (Wirtz et al., 2022). This variant was first identified as a risk factor for familial premature myocardial infarction and although the N700S mutation occurs in the C-terminal calcium binding wire region, protein stability and heparin binding functions are altered by this mutation, suggesting alterations in functions ascribed to other TSP-1 domains: the impact of the N700S mutation on TSP-1Calr/LRP1 signaling is unknown (Zhou et al., 2004; Stenina et al., 2005; Carlson et al., 2008). Links between increased TSP-1 expression and upregulated TGF-β activity suggest a role for TSP-1 in modulating TGF-β activation and possible MMP activity in glaucoma (Wallace et al., 2014; Murphy-Ullrich and Downs, 2015; Keller and Peters, 2022). TSP-1 can activate TGF-β 1-3 isoforms, whereas integrin-dependent TGF-β activation affects only TGF-β1 and 3 activation: since TGF-β2 is a primary factor in glaucoma, this suggests a significant role for TSP-1 in regulating TGF-β1 and 2 activity in glaucoma (Murphy-Ullrich and Downs, 2015; Murphy-Ullrich and Suto, 2018). Post-surgical levels of TSP-1 and TGF-β2 levels in aqueous humor are major independent predictors of trabeculectomy failure associated with fibrotic responses in primary open angle glaucoma patients (Zhu et al., 2019). Another pilot study confirmed elevated aqueous humor TGF-β2 levels in patients with failed trabeculectomy (Gajda-Derylo et al., 2019). It is not known whether aqueous humor levels of these proteins reflect actual signaling at the cellular level or rather are surrogate markers of underlying disease pathogenesis. Despite the focus on TSP-1 as a regulator of TGF-β activation in glaucoma, in contrast, the possible functions of the TSP-1/Calr/LRP1 complex in glaucoma remain unexplored and unknown.

### Factors Involved in Glaucoma Pathogenesis Regulate TSP-1 Expression

#### Mechanical Forces and Mechanotransduction Pathways

It is well documented that TSP-1 is a mechano-responsive gene with numerous examples in studies with both LC and TM cells, as well as in other tissues, such as lung and skin (Kirwan et al, 2005a; Vittal et al., 2005; Warburton and Kaartinen, 2007; Chen et al., 2011). One mechanism for mechano-regulation of TSP-1 expression might occur *via* cellular contractility as recent studies showed that a Rho-kinase inhibitor decreased TSP-1 expression by TM cells, which was associated with decreased cell migration and increased outflow (Shan et al., 2021). Interestingly, in these studies, the LSKL peptide, which prevents TSP-1-mediated latent TGF-β activation, also decreased TSP-1 expression in this system, suggesting that TGF-β regulates TSP-1 expression under these conditions and that TM cell motility occurs downstream of Rho-mediated cytoskeletal contractility. It was not shown whether active Rho kinase regulates TSP-1 expression *via* TGF-β or more directly *via* Rho. It should be noted that the LSKL peptide only prevents TSP-1 binding to the latent TGF-β complex and does not block the action of the hep I peptide on focal adhesion disassembly and F-actin re-organization (unpublished data). Others showed that TSP-1 regulates TGF-β1 induced cell contractility in systemic sclerosis fibroblasts via MEK/ERK signaling, which can be blocked by the LSKL peptide (Chen et al., 2011). We showed that the TSP-1 Calr binding sequence activates ERK and transiently downregulates Rho kinase activity and stimulates cell migration (Orr et al., 2002; Orr et al, 2003a; Orr et al., 2004). The studies by Shan et al. (2021) showing that Rho kinase inhibition downregulated TSP-1 expression resulting in decreased cell migration would be consistent with a role for the TSP-1 Calr binding site in TM cell migration, although this remains to be shown directly and one cannot rule out effects due to TSP-1 mediated TGF-β activation. Moreover, the interplay between Rho regulated TSP-1 expression and TGF-β activation requires further study and these data suggest that the multiplicity of TSP-1 actions in a given tissue in specific context are likely to operate in the context of highly regulated feedback system.

The YAP-TAZ pathway is a key effector in mechanotransduction pathways and it has recently been shown that YAP is increased in LC cells from donors with glaucoma (Murphy et al., 2022). Culturing normal LC cells on stiff matrices increases YAP, expression of collagen and α-smooth muscle actin, and cell proliferation: YAP also regulates myofibroblast differentiation of scleral fibroblasts (Hu et al., 2021; Murphy et al., 2022). In addition, YAP regulates fibrotic activity of TM cells in response to dexamethasone (Liu et al., 2021). Interestingly, TSP-1 expression is regulated by YAP/TAZ and TSP-1 can also regulate YAP/TAZ signaling (Arun et al., 2020; Yamashiro et al., 2020; Arun et al., 2022). In vascular smooth muscle cells under cyclic mechanical stretch, TSP-1 binds to integrin αvβ1 and induces focal adhesion formation to stimulate nuclear shuttling of YAP, which impacts vascular remodeling in injury models (Yamashiro et al., 2020). In contrast, heart endothelial cells expressing TSP-1 had lower levels of unphosphorylated YAP and decreased nuclear YAP when challenged with the parasite *T. cruzi* as compared to endothelial cells from TSP-1 knockout mice (Arun et al., 2020). Furthermore, TSP-1 modulated YAP/β-catenin signaling in this system (Arun et al., 2022). As noted previously, infection of mammalian cells with *T. cruzi* increases TSP-1 expression and TSP-1 binding to parasite Calr on the surface of the trypanosome increases infectivity (Johnson et al., 2012). Interestingly, TSP-1 itself is transcriptionally induced by YAP/TAZ signaling (Shen et al., 2018; Boopathy and Hong, 2019). Exosomes derived from stiff matrices are enriched in TSP-1 and secreted in a YAP-dependent manner, which drives motility and invasiveness of breast cancer cells in a FAK and metalloproteinase manner, although these observations were associated with increased, not decreased, focal adhesions (Patwardhan et al., 2021). Given the importance of mechanotransduction and ECM stiffness in glaucoma (Last et al., 2011; Raghunathan et al., 2013; Powell et al., 2022), the roles of TSP-1 and signaling through its Calr-binding sequence warrant further investigation.

#### Thrombospondin-1 Regulation by Endoplasmic Reticulum Stress

Elevated TGF-β levels and dexamethasone are known factors in the pathogenesis of glaucoma (Flugel-Koch et al., 2004; Kirwan et al, 2005b). Chronically increased ER stress due to aging, oxidative stress, and glucocorticoid (dexamethasone) treatment also plays a role in glaucoma (Peters et al., 2015). ER stress also increases TSP-1 expression in pulmonary tissues subjected to intermittent hypoxia and TSP-1 plays an important role in cardiac hypertrophy-mediated autophagy through the PERK-ATF4 pathway, where recombinant NTD, and to a lesser extent the CTD, co-immunoprecipitated with PERK (Shi et al., 2020; Vanhoutte et al., 2021). Interestingly, TSP-1 is induced in hypertrophic hearts, as are ER stress proteins including Calr: induction of Calr by hypertrophy is attenuated in *Thbs1* -/- mice, suggesting that TSP-1 can regulate expression of Calr, consistent with our unpublished studies (Vanhoutte et al., 2021). Recent studies show that TSP-1 also upregulates ER stress *via* increasing the ATF6-CHOP axis in renal tubular epithelial cells under high glucose conditions (Yue and Du, 2022): unfortunately, neither the active domain of TSP-1 nor the expression of Calr was investigated in these studies. Nonetheless, possible links between TSP-1 signaling and ER stress in glaucoma would be interesting to investigate.

### Thrombospondin-1 Co-Receptors Calr/LRP1 in Glaucoma

Little is known about regulation of the TSP-1 co-receptors, Calr and LRP1, in glaucoma. Calr expression and its localization to the cell surface are increased in ER stress. In a fraction of glaucoma cases in which patients express myocilin with mutations that affect protein folding, aggregation, and retention in the ER [juvenile open-angle, ∼4% of adult onset primary open-angle (Kwon et al., 2009)], mutant myocilin engages pro-glaucoma alterations in cell adhesion and signaling and autophagy (Stone et al., 1997; Liu and Vollrath, 2004; Yan et al., 2020; Sharma and Grover, 2021). The Calr/calnexin chaperones bind to mutant myocilin: chemical chaperones reduce mutant myocilin-Calr binding and reduce ER stress induced cell death (Liu and Vollrath, 2004; Yam et al., 2007; Yan et al., 2020). As dexamethasone can stimulate ER stress in glucocorticoid-induced glaucoma, it is possible that cell surface Calr is also upregulated in this form of glaucoma (Zode et al., 2014). Similarly, chronic conditions associated with glaucoma, such as aging and oxidative stress increase ER stress (Peters et al., 2015). Nonetheless, whether ER stress induces expression of Calr on the cell surface of cells important to TM, scleral, or LC remodeling, where it could mediate signaling *via* TSP-1 ligation, remains unexplored.

There is evidence of extracellular release of Calr from on cytotrophoblast cells under ER stress: given the role of extracellular Calr in scarless wound repair, it would be interesting to know if Calr is increased in the aqueous fluid from glaucomatous TM (Pandya et al., 2020; Iwahashi et al., 2021). Calr plays a role in mediating fibrotic TGF-β signaling through ER-mediated calcium release and NFAT activation and increased levels of Calr have been detected in the medial layer of atherosclerotic rabbits, implicating ER and secreted Calr in ECM remodeling (Gold et al., 2010; Van Duyn Graham et al., 2010; Zimmerman et al., 2013; Owusu et al., 2018).

Similarly, there is little known about modulation of LRP1 activity (endocytosis, signaling) in glaucoma. As LRP1 is a scavenger receptor that mediates endocytosis of TSP-1 and also MMPs, it could play a role in regulating local levels of these proteins. Furthermore, Calr and LRP1 act in concert to signal clearance of apoptotic cells, independent of TSP-1 binding to Calr, an activity that could mediate cellular clearance of damaged TM cells to potentially increase aqueous outflow (Ogden et al., 2001; Gardai et al., 2005; Obeid et al., 2007). However, these TSP-1 independent roles have not been tested in glaucoma and could be potentially important avenues of investigation.

### Localization of the N-Terminal Domain Calr-Binding Sequence in the Glaucomatous Milieu

It is possible that TSP-1 signaling through the NTD Calr-binding sequence plays significant roles in glaucomatous ECM remodeling. The NTD is susceptible to proteolysis by a variety of enzymes, including ADAMTS1, neutrophil elastase, and cathepsin G (Raugi et al., 1984; Rabhi-Sabile et al., 1996; Lee et al., 2006). BMP-1 also cleaves TSP-1 and releases the C-terminal region, which is associated with increased TGF-β activity (Anastasi et al., 2020). Lee et al. (2006) reported the presence of a TSP-1 fragment corresponding to the NTD, both in wound tissue and in wound fluids. The C-terminus determines TSP-1 ECM localization, supporting the idea of released soluble NTD (Adams et al., 2008). This is consistent with our observations of the secreted hep I sequence both in wound fluid and deposited into the wounds of mice with engineered expression of the Calr-binding hep I sequence in the model of the foreign body response described above (Sweetwyne et al., 2010). In a situation of injury, one would expect that the soluble fragment would be the predominant form of TSP-1, although deposition into and release from the provisional or early wound matrix remains a possibility. The influence of “stiff” matrices as found in glaucomatous tissue might also impact deposition and/or release of the TSP-1 NTD.

### Thrombospondin-1/Calr/LRP1 Signaling and Cross-Linked Actin Networks Formation

Despite the role of the TSP-1 Calr binding sequence in stimulating focal adhesion disassembly and re-organization of actin-containing stress fibers, a possible role for TSP-1 in either mediating or antagonizing cross-linked actin networks (CLAN) formation in glaucomatous cells is unknown. The αvβ3 integrin is critical for CLAN formation and fibronectin fibrillogenesis, associated with increased cellular contractility, increased intraocular pressure, and increased TGF-β2 signaling (Filla et al., 2006; Filla et al., 2009; Filla et al., 2011; Filla et al., 2021). Interestingly, the TSP-1 Calr binding sequence activates many of the same pathways (PI3K, ERK, FAK) associated with integrin signaling; however, TSP-1 induces dissociation of α-actinin and vinculin from focal adhesions leading to dissociation of actin stress fibers from focal adhesions without dissociation of αvβ3 integrin clusters, paxillin, or talin (Murphy-Ullrich and Hook, 1989; Greenwood and Murphy-Ullrich, 1998). In the glaucomatous TM, stiffer ECMs are associated with increased stress fibers and CLAN formation: although TSP-1 signaling through Calr/LRP1 can increase collagen matrix, potentially leading to a stiffer ECM, this could be counteracted by induction of focal adhesion disassembly and loss of organized stress fibers that would decrease contractility. It is possible that short-term signaling through TSP-1/Calr/LRP1 would decrease CLAN formation due to focal adhesion disassembly, but support long-term increased CLAN formation due to a denser collagen ECM.

## Discussion: Gaps and Opportunities

**FIGURE 3:**
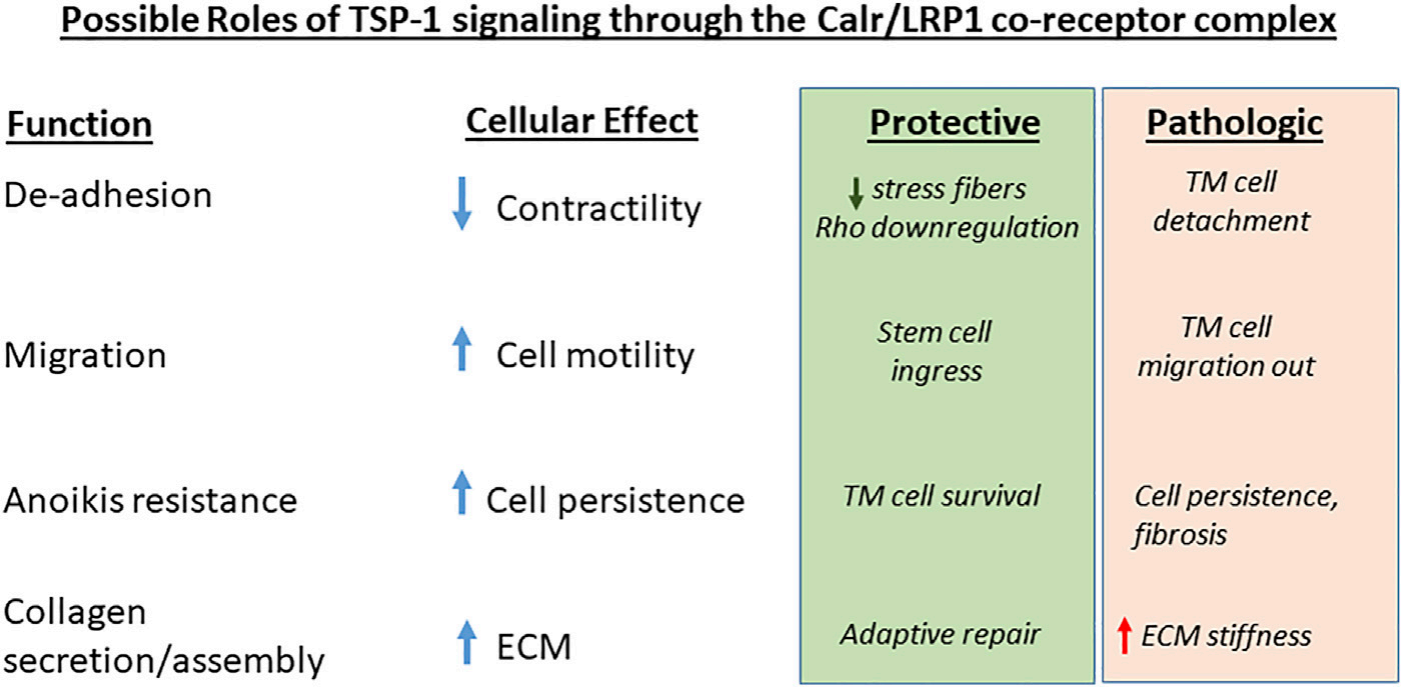
Possible roles of TSP-1/Calr/LRP1 signaling in glaucoma. The four major functions ascribed to TSP-1 signaling through the Calr/LRP1 receptor co-complex and their cellular effects are designated. Potential beneficial or pathologic effects of these activities in glaucoma cell responses to injury and ECM remodeling are indicated.

Here we have focused specifically on the roles of the TSP-1/Calr binding sequence and its signaling in concert with LRP1 in regulating focal adhesion disassembly and cell motility, promotion of cell survival under de-adhesive conditions, and collagen matrix assembly**.** The importance of these TSP-1 functions in TM, LC, and scleral remodeling in glaucoma has been largely unexplored. It is not clear whether these functions might contribute to the pathogenesis of glaucoma through stimulation of cell migration, cell persistence, and collagen deposition (Figure 3). The ability of the TSP-1/Calr/LRP1 axis to promote collagen ECM assembly suggests ECM stiffness might be increased *via* this pathway, which could increase pathologic remodeling. However, the disruption of focal adhesion connections to actin-containing stress fibers suggests that TSP-1/Calr/LRP1 signaling would decrease cellular contractility and be protective against glaucoma. Increased cell survival and persistence of ECM-producing cells in response to injury is associated with fibrotic remodeling, suggesting that TSP-1 induced anoikis resistance might increase survival of fibrosis promoting cells, especially during migration (Thannickal and Horowitz, 2006). Alternately, TM cell loss is characteristic of glaucoma and TSP-1 promotion of cell survival might be protective. Some think that TM cell loss is partially due to TM cell migration out of the outflow tract to the Schlemm’s canal lumen after injury, which potentially could be exacerbated by TSP-1 signaling through Calr/LRP1 (Calthorpe et al., 1991; Hogg et al., 1995; Zhou et al., 1999; Hogg et al., 2000). On the other hand, TSP-1 signaling could attract stem cells to sites of injury or surgical intervention and enhance repair (Kelley et al., 2009; Yun et al., 2016).

Only direct studies *in vitro* and in relevant animal models can address these questions. If data suggest that TSP-1 signaling through this pathway is deleterious, one could deliver an antagonist peptide of the Calr TSP-1 binding sequence (CRT19.36) (Sweetwyne et al., 2010). Alternately, the hep I peptide (aa 17–35) could be used to mimic and augment any beneficial TSP-1 effects. Similar approaches using intraocular delivery of collagen mimetic peptides to repair retinal ganglion cells have been shown to have benefit in rodent models (McGrady et al., 2021; Ribeiro et al., 2022).
